# The Polarization States of Microglia in TBI: A New Paradigm for Pharmacological Intervention

**DOI:** 10.1155/2017/5405104

**Published:** 2017-02-01

**Authors:** Hangzhe Xu, Zhijiang Wang, Jianru Li, Haijian Wu, Yucong Peng, Linfeng Fan, Jingyin Chen, Chi Gu, Feng Yan, Lin Wang, Gao Chen

**Affiliations:** ^1^Department of Neurosurgery, Second Affiliated Hospital, School of Medicine, Zhejiang University, Jiefang Road 88th, Hangzhou 310016, China; ^2^Department of Neurosurgery, Sir Run Run Shaw Hospital, School of Medicine, Zhejiang University, Qingchun East Road 3rd, Hangzhou 310016, China

## Abstract

Traumatic brain injury (TBI) is a serious medical and social problem worldwide. Because of the complex pathophysiological mechanisms of TBI, effective pharmacotherapy is still lacking. The microglial cells are resident tissue macrophages located in the brain and have two major polarization states, M1 phenotype and M2 phenotype, when activated. The M1 phenotype is related to the release of proinflammatory cytokines and secondary brain injury, while the M2 phenotype has been proved to be responsible for the release of anti-inflammation cytokines and for central nervous system (CNS) repair. In animal models, pharmacological strategies inhibiting the M1 phenotype and promoting the M2 phenotype of microglial cells could alleviate cerebral damage and improve neurological function recovery after TBI. In this review, we aimed to summarize the current knowledge about the pathological significance of microglial M1/M2 polarization in the pathophysiology of TBI. In addition, we reviewed several drugs that have provided neuroprotective effects against brain injury following TBI by altering the polarization states of the microglia. We emphasized that future investigation of the regulation mechanisms of microglial M1/M2 polarization in TBI is anticipated, which could contribute to the development of new targets of pharmacological intervention in TBI.

## 1. Introduction

Traumatic brain injury is a major health problem worldwide [1, 2]. The pathophysiological mechanisms of TBI are complex and unclear, and effective pharmacotherapies for TBI patients remain lacking. Thus, further elucidation of the pathophysiological mechanisms of TBI is warranted, and it could help to develop new targets of pharmacological intervention for TBI.

Neuroinflammation, which includes activation of local microglia and recruitment of other immune cells from the blood, as well as production of inflammatory cytokines, plays an important role in the pathophysiology of TBI [3]. On the one hand, neuroinflammation is detrimental and contributes to brain injury following TBI; on the other hand, neuroinflammation is necessary for the clearance of harmful substances, such as cell debris, after TBI [4]. Therefore, elucidation of the role and the underlying molecular mechanisms of neuroinflammation in TBI pathology is extremely vital for presenting potential new therapeutic targets for TBI.

The microglial cells are the resident macrophage cells of the brain [5], and they can activate rapidly in response to pathological changes in the central nervous system (CNS) [6, 7], for example, traumatic/ischemic brain injury or subarachnoid hemorrhage (SAH). In recent years, researchers have discovered two polarization states of microglial cells when they are activated, the M1 phenotype and the M2 phenotype [8–11], exactly like macrophages in nonneuronal tissues [12]. There is a large difference between the roles of the two phenotypes of activated microglial cells: the M1 phenotype is related to the release of several proinflammatory cytokines, such as tumor necrosis factor-alpha (TNF-*α*), while the M2 phenotype has been proved to be responsible for the release of anti-inflammatory cytokines, such as interleukin-10 (IL-10), and for neural regeneration processes, such as neurogenesis, angiogenesis, oligodendrogenesis, and remyelination [10]. Thus, how to alter the M1/M2 ratio to improve functional recovery after TBI has become a new pharmacological therapeutic direction in TBI. In this review, we aimed to summarize the current knowledge about the pathological significance of microglial M1/M2 polarization in the pathophysiology of TBI. In addition, we reviewed several drugs that have provided neuroprotective effects against brain injury following TBI by altering the polarization states of the microglia.

## 2. Microglial Phenotypes and Pathological Significance in TBI

In the parenchyma of the CNS, microglia constitute about 0.5% to 16.6% of all cells, especially in the white matter [13]. These microglial cells can remove cell debris and toxic substances, thus maintaining the homeostasis of the CNS. When insult occurs, the microglial cells activate rapidly, change their morphology into a motile “amoeboid” state, proliferate, and migrate into the damaged regions, and they release a variety of cytokines depending on the polarization states [14, 15]. Nowadays, there is a growing number of papers discussing the topic of phenotypes of microglia. The keyword “microglia phenotypes” return over 1,700 hits from PubMed in later 2016, and more than half of them were published in recent four years. Thus, exploring of the mechanisms for microglia/macrophage phenotype shift is becoming a meaningful topic.

**Figure 1:**
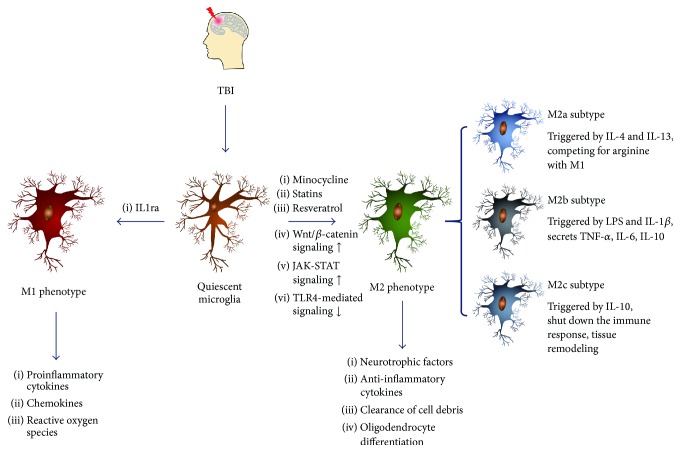
Two distinct polarization states of activated microglial cells and the three different subtypes of M2 phenotype microglia.

Two distinct polarization states of the activated microglial cells have been discovered, the M1 phenotype and the M2 phenotype, depending on particular microenvironments [16]. The M1 phenotype microglia are induced by proinflammatory molecules, such as lipopolysaccharide (LPS) or interferon-gamma (IFN-*γ*). This phenotype closely matches the classical activation stage and secretes high levels of proinflammatory cytokines, such as IFN-*γ*, TNF-*α*, IL-1*β*, chemokines, and reactive oxygen species (ROS), which are essential for host defense [17]. They also secrete a low level of IL-10 [18]. The M2 phenotype microglia are subdivided into three subtypes: M2a, M2b, and M2c. Each of these subtypes has different trigger factors and phenotypic markers. The M2a subtype, which can be triggered by IL-4 and IL-13, is the first alternative activation stage of the microglia. It can act as an anti-inflammatory microglia subtype compared to M1 phenotype by competing for arginine, a nitrogen pool for the production of reactive nitrogen species during M1 phase [19]. The M2b subtype is a mixed activation state of microglia, it produces pro- and anti-inflammatory cytokines, such as TNF-*α*, IL-6, and IL-10, and it can be triggered by treating with LPS and IL-1*β* concurrently or with activated IgA complexes [19, 20]. The M2c subtype can be triggered by IL-10; it can shut down the immune response of the microglia [19], and it appears to play a role in tissue remodeling and matrix deposition [17] (Figure 1). The neuroprotective effects of M2 phenotype microglia have been well-studied, including the secretion of neurotrophic factors and anti-inflammatory cytokines and the clearance of cell debris through phagocytosis [10, 14]. Moreover, M2 microglia/macrophages have been found to play essential roles in driving oligodendrocyte differentiation toward the process of remyelination in the central nervous system, probably by contributing transforming growth factor-*β* (TGF-*β*) superfamily member activin-A to the remyelinating lesion environment [21].

Because different phenotypes of microglia appear and function in different phases, it is necessary to determine the time evolution of each subtype of microglial cells after TBI. However, this process has not yet been completely learned, and the experimental results have seemed different between in vitro and in vivo models. Several years ago in an in vitro experiment, researchers found that “acutely activated microglia” could reduce neural precursor cell survival and prevent neuronal differentiation, while “chronically activated microglia” could facilitate neural differentiation and cell survival [22]. Some experimental studies have supported that the “chronically activated microglia” seem to be M2 phenotype microglia and not M1. For example, there is an alteration in cytokine production. In a prolonged in vitro experiment, when exposed to LPS, the proinflammatory cytokines IL-1*α*/*β*, IL-6, and TNF-*α* secreted by microglia were strongly decreased, while the anti-inflammatory cytokine IL-10 and the immunomodulatory prostaglandin E2 (PGE2) increased greatly, indicating that the chronically activated M2 phenotype microglia might play a beneficial role in neuronal differentiation and cell survival [22]. In addition, in normal wound and spinal cord injury (SCI) healing, the M1 and M2a types seem to be the primary subtypes that start immediately after brain injury, and then they are gradually replaced by the M2b and M2c subtypes after 3 dpi (days after injury), indicating the start of the proliferation phase [23]. However, with in vivo TBI models, the findings have been different. Jin et al. [24] found that CD206(+)/CD11b(+) M2 phenotype microglia were increased at 1 week after controlled cortical impact (CCI), whereas CD86(+)/CD11b(+) M1 phenotype microglia were increased at 4 weeks. In a mouse model of TBI, transient M2 phenotype microglia were the initial phenotype of activated microglia in the acute stage of brain insult and peaked at 5 days after injury, but the M1 phenotype microglia remained elevated until at least 14 days [25]. Recently, another in vivo experiment demonstrated that the M2 phenotype microglia were upregulated transiently and then were replaced by M1 or a mixed transitional phenotype at 7 days after injury [26]. Actually, activated microglia could be observed in the injured cortex even 1 year after injury in a mouse CCI model, in association with lesion expansion and neurodegeneration [27]. The conflict of chronic microglial phenotypes between in vitro and in vivo models might be due to the complex signaling events surrounding microglial cells in vivo, and exploring the mechanisms underlying this phenomenon could help us to find new targets of pharmacological intervention in TBI. In humans, using the positron emission tomography (PET) ligand [11C](R)PK11195 (PK), researchers demonstrated that increased microglial activation could persist for 17 years after TBI [28]. Another study of human brain samples after TBI found that the reactive microglia could exist for up to 18 years after trauma [29]. Although the phenotype of these microglia cells has not been clarified, these activated microglia indicate a chronic inflammatory response after brain injury.

In addition, it is important to know that this classification system only represents two major extreme types of activated microglia. Currently, this paradigm that simply divide the activated microglia into M1/M2 phenotypes has been challenged. Unlike the simple microenvironments in the in vitro experiments, the complex signaling events after tissue injury in vivo can lead the microglia/macrophage to a complex response or perhaps mixed phenotypes [30], which is consistent with previous studies focused on single-cell level. In mouse CCI model, microglia/macrophages could concurrently express both M1 and M2 phenotypic markers on the same cell in multiple time points [31]. This phenomenon has also been observed in other diseases such as multiple sclerosis [32] and cutaneous squamous cell carcinoma [33]. Also, the activated microglia population can change from an early “healthy” M2 phenotype into a “sick” M1 phenotype and exist for a very long time [24, 31, 34]. This point of view has changed our treatment concept of TBI and other brain insults in recent years. Therapies should no longer focus only on suppressing microglia/macrophage activation, but they should pay greater attention to the ratio of M1/M2 phenotype of microglia to decrease the harmful effects of neuroinflammation [10, 14, 35].

## 3. Polarization States of Microglia in TBI: Potential Therapeutic Targets for Pharmacological Intervention

So far, many anti-inflammation drugs have been discovered to manage the neuroinflammation process after TBI. Although some of these drugs have shown neuroprotective effects in animal models, none have succeeded in clinical trials, perhaps due to the strict treatment time window and the heterogeneity of subtypes of TBI. Microglial cells are the target of many anti-inflammation strategies, and their phenotypes are of great significance in TBI treatment. As mentioned above, the dominant phenotype of activated microglia could shift from acutely activated M2 to chronically activated M1 after TBI [26]. This time dependent property of microglia activation indicates that M2 phenotype microglia may have more potential in clearing cell debris in the early stage of TBI, and M1 phenotype microglia may have relationship with chronic neuroinflammation [35]. So, anti-inflammation strategies that targeted whole microglia system might not be the best answer. An increasing number of studies trying to regulate the ratio of M1 and M2 phenotype microglia have already got promising effects after brain injury, which made the polarization states of microglia a new paradigm for pharmacological intervention. Strategies inhibiting the M1 phenotype and promoting the M2 phenotype of microglial cells could alleviate cerebral cell damage and improve neurological function recovery in variety of brain injury animal models such as CCI [18, 36] and middle cerebral artery occlusion (MCAO) [37, 38]. These efforts pay more attention on later neurogenesis, which directly leads to better long-term prognosis. Thus, developing drugs that can modulate the immune system through altering the M1/M2 ratio is a promising pharmacological intervention in TBI treatment.

However, although the phenotypes and roles of microglia have been known for several years, many drugs with the effect of inhibiting microglial activation are still regarded as having the effect of anti-inflammation and many studies that have evaluated microglia activation only using pan-microglial markers such as Iba-1 or Cd11b [3]. In fact, total microglia activation inhibition cannot represent the anti-inflammation effect, and the therapeutic purpose is far more than anti-inflammation. In this section, we review several drugs that might provide neuroprotective effects after TBI by changing microglia polarization states, and we discuss the potential mechanisms underlying them. These mechanisms include enhanced Wnt/*β*-catenin signaling and JAK-STAT signaling, inhibition of TLR4-mediated signaling, and p38 MAPK-dependent PPAR*γ* activation.

### 3.1. Minocycline

Minocycline is a second-generation tetracycline with a variety of nonantibiotic biological effects, such as neuroprotection in experimental models of TBI, ischemia, and neurodegenerative diseases [39]. The anti-inflammation effect is the most well-known advantage of the neuroprotective effects of minocycline. A series of studies have demonstrated that minocycline can inhibit microglial activation, using pan-microglial markers in TBI, SCI, SAH, and cerebral ischemia [40–45]. Although there is a large amount of data showing this anti-inflammation effect is mostly mediated by microglia, the molecular targets still need to be discovered. One possible explanation is that minocycline may regulate microglial activation through inhibition of poly(ADP-ribose) polymerase (PARP), since minocycline contains an aromatic ring-linked carboxamide group just as other competitive PARP inhibitors [46], and PARP regulates the NF-*κ*B driven transcription and microglia activation [47].

Despite the huge data of microglia inhibition effect, a few studies have demonstrated the function of minocycline in changing the M1/M2 ratios of microglial cells. Kobayashi and colleagues found that minocycline selectively inhibited M1 but not M2 microglia in a mouse amyotrophic lateral sclerosis (ALS) model and in primary cultured microglial cells [48]. Another study using minocycline-loaded nanoparticles in SCI drew a similar conclusion [49]. In a rat model of depression, chronic administration of minocycline decreased not only the expression of the pan-microglial marker CD11b but also the M1 proinflammatory cytokine IL-1*β* in sham spinal nerve ligation (SNL) animals. The expression of the M2 microglia marker MRC2 and of IL-10 and IL-1*β* was increased in the prefrontal cortex of olfactory bulbectomized-SNL rats, which indicated that chronic minocycline administration had an effect on altering M1/M2 microglia markers [50]. Recently, in a cerebral ischemia model, researchers discovered that treatment with minocycline could significantly decrease the levels of TNF-*α* and IL-1*β* and increase the levels of TGF-*β*, IL-10, and YM1 [51]. These results indicated that minocycline might also have the alternate effect of microglia/macrophage activation in TBI, and further research must be undertaken to discover it.

### 3.2. Etanercept

Etanercept is a biologic TNF antagonist, and it has been proved to have anti-inflammatory effects by inhibiting brain TNF-*α* [15]. Considering its molecular weight, it is too large to pass the blood-brain barrier (BBB); therefore, it cannot reach therapeutic concentrations in the cerebrospinal fluid in theory [52]. However, experiments have confirmed that etanercept can penetrate into contused brain tissues for unknown reasons and can play a neuroprotective role in a series of diseases, such as TBI [53, 54], SCI [55, 56], and ischemic brain injury [57, 58]. The functions of etanercept in TBI are not only to inhibit microglial activation [15] but also to reduce early overexpression of TNF-*α* in microglia [54] and to stimulate the newly formed neurogenesis [53]. Thus, etanercept can attenuate the effects of M1 phenotype microglia and can have neural regeneration effects, like M2 phenotype microglia [18]. These experiments showed the potential effects of etanercept in altering the M1/M2 ratio and promoting neural regeneration. Furthermore, treatment with perispinal etanercept of chronic stroke and TBI patients could produce clinical improvement, even a decade later [59]. The therapeutic efficacy of etanercept in TBI is worth studying.

### 3.3. Statins

Statins, known as 3-hydroxy-3-methylglutaryl coenzyme A (HMG-CoA) reductase inhibitors, are well known for the effects of lowering serum cholesterol level and decreasing the risk of cardiovascular events [60]. Despite their cholesterol-lowering function, the anti-inflammatory effects of statins have gained recognition in recent years. Because of their pleiotropicity, statins have been used in the management of ischemic stroke [61], neurodegenerative diseases [62, 63], and even chronic subdural hematoma [64]. In TBI management, simvastatin has the effects of attenuating microglia and astrocyte activation, decreasing proinflammatory cytokines such as IL-1*β*, IL-6, and TNF-*α* [65], suppressing neuronal cell apoptosis [66], and inducing angiogenesis [67]. Although the microglia inhibition effects of statins have been proved, the mechanisms are still in exploration. In cultured microglia cells, rosuvastatin strongly inhibited microglia proliferation and adhesion and additionally increased the expression of several anti-inflammatory genes such as* Ccl2* and* Cxcl1*, which were implicated in microglia recruitment [68]. Another in vivo study suggests that simvastatin can exert analgesic effects by attenuating spinal microglial activation through interruption of microglial RhoA translocation and p38 MAPK activation [69].

To our knowledge, there has been no direct evidence of statins altering the M1/M2 ratio of microglia, but some studies have suggested a positive answer. In an in vitro experiment, simvastatin could play an anti-inflammation role by enhancing the switching of the M1 macrophage phenotype to the M2 macrophage phenotype [70]. Atorvastatin also has shown similar effects in myocardial infarction [71] and might promote circulating monocyte differentiation into M2 phenotype macrophages via p38 MAPK-dependent PPAR*γ* activation [72]. The effects of statins on microglial activation and differentiation in TBI deserve further research.

### 3.4. Resveratrol

Resveratrol, a stilbene formed in many edible plants as a reaction to fungal infection, has been proved effective in cancer, heart diseases, and a series of nervous system diseases in vitro and in vivo [73, 74], also including ischemic stroke [74, 75], neurodegenerative diseases [76], TBI [74, 77], and SAH [78]. There are many potential pathways for the neuroprotective effects of resveratrol, such as activation of the silent mating type information regulation 2 homolog 1 (SIRT1), AMP-activated kinase (AMPK), nuclear factor erythroid 2 (Nrf2) pathways [74], and inhibition of the NF-*κ*B, ERK, and JNK/MAPK pathways [79, 80]. The inhibition of the microglial activation of resveratrol occurs not only in TBI models [81] but also in other in vitro and in vivo models [82–84], possibly by activating an SOCS-1-mediated signaling pathway [85], and it might be associated with neurogenesis [86]. As a natural modulator of microglial activity, resveratrol has the ability to counteract the excessive response of activated M1 microglia [87]. In a BV2 microglia cell line of hypoxia injury model, resveratrol suppressed the mRNA expression of TNF-*α* and promoted the mRNA expression of IL-10 [88], suggesting its ability to change microglia phenotypes. Another experiment using LPS-stimulated microglia drew the same conclusion, and the JAK-STAT signaling pathway might be involved in this process [89]. Hence, the prospective value of resveratrol in TBI is worth being studied further.

### 3.5. MGluR5 Agonist and Positive Allosteric Modulator

In recent years, the metabotropic glutamate receptor 5 (mGluR5) selective agonist (RS)-2-chloro-5-hydroxyphenylglycine (CHPG) and the positive allosteric modulator (PAM) VU0360172 have attracted increasing attention in the treatment of various brain insults. Both CHPG and VU0360172 could significantly reduce the numbers of activated microglia and improve neurological function in a rat endovascular perforation model of SAH [90]. CHPG could limit neuroinflammation and improve functional recovery even a month later after TBI [91, 92], and it significantly decreased the levels of proinflammatory cytokines and increased the expression of anti-inflammatory cytokines after SO_2_ treatment in BV2 microglial cells [93]. This anti-inflammatory effect might be mediated by G-protein signal transduction pathway, including activation of the phospholipase C-protein kinase C signal transduction system [94]. However, because of the weak potency and brain permeability of CHPG, researchers have found another more efficient mGluR5 PAM, VU0360172, and its ability to shift the M1/M2 microglial activation balance towards an M2 phenotype in vivo and in vitro has been shown [95]. An increasing number of studies have reported the protective effects of mGluR5 agonists and PAMs, making this young drug class a promising agent in TBI therapy.

### 3.6. Gp91ds-tat

Gp91ds-tat is a selective nicotinamide adenine dinucleotide phosphate oxidase (NOX-2) inhibitor. The neuroprotective effect of gp91ds-tat was first discovered in cerebrovascular dysregulation associated with increasing age [96, 97]. A more recent study showed that gp91ds-tat could significantly improve functional recovery and reduce inflammation in an SCI model, accompanied by a reduction in M1 microglia phenotype markers [98]. It seems alterations in microglia polarization and NOX activity could influence each other [98–100]. In a mouse CCI model, gp91ds-tat significantly reduced neuron damage and edema [101], and delayed gp91ds-tat treatment (24 h post injury) could alter the M1/M2 balance to the anti-inflammatory M2 phenotype, reducing oxidative damage in neurons and cognitive function deficits [26, 102]. Therefore, gp91ds-tat could have promising therapeutic effects in TBI treatment.

### 3.7. Rosiglitazone

As a peroxisome-proliferator-activated receptor- (PPAR-) *γ* agonist, rosiglitazone is not only an antidiabetic drug but also a neuroprotective agent, and it has shown various effects in treating brain ischemia [103], TBI [104], and SAH [105]. A study demonstrated rosiglitazone's ability to attenuate microglia/macrophage activation and neuronal loss after TBI [104]. In mouse models of focal cerebral ischemia and progressive Parkinson's disease, rosiglitazone showed the ability to promote microglial M2 polarization [103, 106]. Another PPAR-*γ* agonist pioglitazone has also been reported to decrease the M1/M2 ratio in experimental Alzheimer's disease [107], but the relationship between PPAR-*γ* agonists and microglia phenotype switching is still not clear. Since studies about rosiglitazone's function in microglia have been very limited, the effects of rosiglitazone in TBI still require exploration.

### 3.8. Azithromycin

Many macrolide antibiotics might have neuroprotective effects. Among them, azithromycin is an extraordinary drug with the effect of reducing infarct volume, decreasing brain edema, and increasing neurological deficit scores in acute ischemic damage [108]. Additionally, azithromycin had the effect of altering the macrophage phenotype from proinflammatory M1 to alternatively activated M2 cells [109, 110], probably by inhibition of TLR4-mediated signaling [111] or activation of activator protein-1 and impairment of lysosomal functions [112]. This effect was observed not only in chronic obstructive pulmonary disease [110] but also in ischemic brain injury [113] and spinal cord injury [114] at a dose of approximately 150 mg/kg. Whether it will be effective in altering microglia phenotypes in TBI treatment remains to be determined.

### 3.9. Alpha-7 Nicotinic Acetylcholine Receptor Agonists

TBI-induced deficits in alpha-7 nicotinic acetylcholine receptor (nAchR) expression were found to play a role in cognitive impairment as early as 2003 [115]. In recent years, nAchR has been receiving attention again. *α*-7 nAchR agonist, PNU-282987, could attenuate early brain injury in SAH rats [116] and could reduce peripheral inflammation and BBB disruption in TBI mice [117]. Another *α*-7 nAchR agonist, PHA568487, could attenuate neuroinflammation, oxidative stress, and brain injury in stroke and bone fracture mice, probably by decreasing the number of M1 phenotype microglia/macrophages and by increasing M2 phenotype microglia/macrophages [118, 119]. Future experiments are needed to determinate whether such drugs also have the same effects in TBI.

### 3.10. Interleukin-1 Receptor Antagonist

Interleukin-1 receptor antagonist (IL1ra) is a competitive antagonist of the interleukin-1 type-1 receptor (IL-1R). Researchers identified the effects of IL1ra in improving recovery and delayed cytokine induction in ischemic brain injury and TBI a dozen years ago [120, 121]. Several experiments on IL-1*β* antibody in TBI have shown similar conclusions [122, 123]. In 2014, a phase II, randomized, controlled trial of recombinant human IL1ra in severe TBI reported promising data using this agent in the modification of neuroinflammatory response [124]. Recently, using Partial Least Squares Discriminant Analysis, researchers have found a “counterintuitive” effect of rhIL1ra on microglia, namely, that rhIL1ra treatment could increase microglial activation following severe TBI and could bias the microglial responses to the M1 phenotype but not the M2 phenotype following human TBI [125]. This result has caused us to rethink the theory of dividing cytokines into “pro-” and “anti-” inflammatory subtypes simplistically in brain insults such as TBI.

### 3.11. Cell Therapy

Cell therapy has been proved to be effective in many kinds of brain injury, including TBI [126]. Nowadays researchers have found that the neuroprotective mechanism of cell therapy is limited to not only neuronal replacement, but also immunomodulation. Mosher et al. showed that transplanted neural progenitor cells (NPCs) could secrete a variety of signaling proteins which have the capacity to modulate microglia functions and activity [127]. Liu et al. described that NPCs and microglia could be significantly affected by each other's presence in an allogeneic coculture model, and NPCs might have the ability of regulating the phagocytic activity of microglia [128]. These results have led to several recent studies about cell therapy's effect in mediating the microglial/macrophage phenotypes after TBI. For example, intravenous delivery of multipotent adult progenitor cells (MAPC) and intracerebral injection of human neural stem cells (NSCs) after experimental TBI can both demonstrate a decrease in M1/M2 ratio [129, 130]. Thus, modulation of M1/M2 ratio through cell therapy could be one of the therapeutic methods after TBI.

## 4. Conclusion

So far, there is still no management providing a strong benefit in TBI, so it is important to find therapeutic drugs with promising new mechanisms after brain damage. The neuroinflammation process, which could serve a dual function, is of great importance after TBI. Different phenotypes of activated microglial cells could play different roles in the neuroinflammation process, so finding new drugs that can alter the M1/M2 ratio of activated microglial cells could be a promising approach to decrease neuroinflammation damage and to improve outcomes. In this review, we summarized the current knowledge about the pathological significance of microglial M1/M2 polarization in the pathophysiology of TBI, and we listed several drugs with neuroprotective effects against TBI by altering the polarization states of microglial cells.

However, some limitations in the current research should be noticed. First, the methods for evaluating the effects of drugs on microglial cells must keep up with the pace; simply inhibiting the activation of microglial cells cannot replace anti-inflammation and neuroprotection. Second, the appearing time periods of the subtypes of microglial cells and the changes in the microenvironment surrounding these cells after TBI must be more deeply explored to discover the best therapeutic time window for the aforementioned drugs. Third, most of the current experiments in pharmacological intervention have focused on the phenomenon of microglial phenotype changes, while studies of mechanisms have been fewer. Future investigations should focus on the regulation mechanisms of microglial M1/M2 polarization and develop new targets of pharmacological intervention in TBI, thus providing new hope for TBI management.

## References

[1] Rosenfeld J. V., Maas A. I., Bragge P., Morganti-Kossmann M. C., Manley G. T., Gruen R. L. (2012). Early management of severe traumatic brain injury. *The Lancet*.

[2] Maas A. I., Stocchetti N., Bullock R. (2008). Moderate and severe traumatic brain injury in adults. *The Lancet Neurology*.

[3] Bergold P. J. (2016). Treatment of traumatic brain injury with anti-inflammatory drugs. *Experimental Neurology*.

[4] Russo M. V., McGavern D. B. (2016). Inflammatory neuroprotection following traumatic brain injury. *Science*.

[5] Helmut K., Hanisch U.-K., Noda M., Verkhratsky A. (2011). Physiology of microglia. *Physiological Reviews*.

[6] Kreutzberg G. W. (1996). Microglia: a sensor for pathological events in the CNS. *Trends in Neurosciences*.

[7] Chiu C., Liao Y., Yang L. (2016). Neuroinflammation in animal models of traumatic brain injury. *Journal of Neuroscience Methods*.

[8] Xiong X.-Y., Liu L., Yang Q.-W. (2016). Functions and mechanisms of microglia/macrophages in neuroinflammation and neurogenesis after stroke. *Progress in Neurobiology*.

[9] Orihuela R., McPherson C. A., Harry G. J. (2016). Microglial M1/M2 polarization and metabolic states. *British Journal of Pharmacology*.

[10] Loane D. J., Kumar A. (2016). Microglia in the TBI brain: the good, the bad, and the dysregulated. *Experimental Neurology*.

[11] Ma Y., Wang J., Wang Y., Yang G.-Y. (2016). The biphasic function of microglia in ischemic stroke. *Progress in Neurobiology*.

[12] Sica A., Mantovani A. (2012). Macrophage plasticity and polarization: in vivo veritas. *Journal of Clinical Investigation*.

[13] Mittelbronn M., Dietz K., Schluesener H. J., Meyermann R. (2001). Local distribution of microglia in the normal adult human central nervous system differs by up to one order of magnitude. *Acta Neuropathologica*.

[14] Kim J.-Y., Kim N., Yenari M. A. (2015). Mechanisms and potential therapeutic applications of microglial activation after brain injury. *CNS Neuroscience and Therapeutics*.

[15] Chio C.-C., Lin J.-W., Chang M.-W. (2010). Therapeutic evaluation of etanercept in a model of traumatic brain injury. *Journal of Neurochemistry*.

[16] Gordon S., Taylor P. R. (2005). Monocyte and macrophage heterogeneity. *Nature Reviews Immunology*.

[17] Colton C. A. (2009). Heterogeneity of microglial activation in the innate immune response in the brain. *Journal of Neuroimmune Pharmacology*.

[18] Chio C.-C., Lin M.-T., Chang C.-P. (2015). Microglial activation as a compelling target for treating acute traumatic brain injury. *Current Medicinal Chemistry*.

[19] Bell-Temin H., Culver-Cochran A. E., Chaput D. (2015). Novel molecular insights into classical and alternative activation states of microglia as revealed by stable isotope labeling by amino acids in cell culture (SILAC)-based proteomics. *Molecular and Cellular Proteomics*.

[20] Mosser D. M. (2003). The many faces of macrophage activation. *Journal of Leukocyte Biology*.

[21] Miron V. E., Boyd A., Zhao J.-W. (2013). M2 microglia and macrophages drive oligodendrocyte differentiation during CNS remyelination. *Nature Neuroscience*.

[22] Cacci E., Ajmone-Cat M. A., Anelli T., Biagioni S., Minghetti L. (2008). In vitro neuronal and glial differentiation from embryonic or adult neural precursor cells are differently affected by chronic or acute activation of microglia. *GLIA*.

[23] Gensel J. C., Zhang B. (2015). Macrophage activation and its role in repair and pathology after spinal cord injury. *Brain Research*.

[24] Jin X., Ishii H., Bai Z., Itokazu T., Yamashita T. (2012). Temporal changes in cell marker expression and cellular infiltration in a controlled cortical impact model in adult male C57BL/6 mice. *PLoS ONE*.

[25] Wang G., Zhang J., Hu X. (2013). Microglia/macrophage polarization dynamics in white matter after traumatic brain injury. *Journal of Cerebral Blood Flow and Metabolism*.

[26] Kumar A., Alvarez-Croda D., Stoica B. A., Faden A. I., Loane D. J. (2016). Microglial/macrophage polarization dynamics following traumatic brain injury. *Journal of Neurotrauma*.

[27] Loane D. J., Kumar A., Stoica B. A., Cabatbat R., Faden A. I. (2014). Progressive neurodegeneration after experimental brain trauma: association with chronic microglial activation. *Journal of Neuropathology and Experimental Neurology*.

[28] Ramlackhansingh A. F., Brooks D. J., Greenwood R. J. (2011). Inflammation after trauma: microglial activation and traumatic brain injury. *Annals of Neurology*.

[29] Johnson V. E., Stewart J. E., Begbie F. D., Trojanowski J. Q., Smith D. H., Stewart W. (2013). Inflammation and white matter degeneration persist for years after a single traumatic brain injury. *Brain*.

[30] Martinez F. O., Gordon S. (2014). The M1 and M2 paradigm of macrophage activation: time for reassessment. *F1000Prime Reports*.

[31] Morganti J. M., Riparip L.-K., Rosi S. (2016). Call off the dog(ma): M1/M2 polarization is concurrent following traumatic brain injury. *PLoS ONE*.

[32] Vogel D. Y. S., Vereyken E. J. F., Glim J. E. (2013). Macrophages in inflammatory multiple sclerosis lesions have an intermediate activation status. *Journal of Neuroinflammation*.

[33] Pettersen J. S., Fuentes-Duculan J., Suárez-Farĩas M. (2011). Tumor-associated macrophages in the cutaneous SCC microenvironment are heterogeneously activated. *Journal of Investigative Dermatology*.

[34] Hu X., Li P., Guo Y. (2012). Microglia/macrophage polarization dynamics reveal novel mechanism of injury expansion after focal cerebral ischemia. *Stroke*.

[35] Jin X., Yamashita T. (2016). Microglia in central nervous system repair after injury. *Journal of Biochemistry*.

[36] Gao C., Qian Y., Huang J. (2016). A three-day consecutive fingolimod administration improves neurological functions and modulates multiple immune responses of CCI Mice. *Molecular Neurobiology*.

[37] Li D., Wang C., Yao Y. (2016). mTORC1 pathway disruption ameliorates brain inflammation following stroke via a shift in microglia phenotype from M1 type to M2 type. *The FASEB Journal*.

[38] He Y., Ma X., Li D., Hao J. (2016). Thiamet G mediates neuroprotection in experimental stroke by modulating microglia/macrophage polarization and inhibiting NF-*κ*B p65 signaling. *Journal of Cerebral Blood Flow & Metabolism*.

[39] Garrido-Mesa N., Zarzuelo A., Gálvez J. (2013). Minocycline: far beyond an antibiotic. *British Journal of Pharmacology*.

[40] Adembri C., Selmi V., Vitali L. (2014). Minocycline but not tigecycline is neuroprotective and reduces the neuroinflammatory response induced by the superimposition of sepsis upon traumatic brain injury. *Critical Care Medicine*.

[41] Haber M., Abdel Baki S. G., Grin'kina N. M. (2013). Minocycline plus N-acetylcysteine synergize to modulate inflammation and prevent cognitive and memory deficits in a rat model of mild traumatic brain injury. *Experimental Neurology*.

[42] Li J., Chen J., Mo H. (2016). Minocycline protects against NLRP3 inflammasome-induced inflammation and P53-associated apoptosis in early brain injury after subarachnoid hemorrhage. *Molecular Neurobiology*.

[43] Marchand F., Tsantoulas C., Singh D. (2009). Effects of Etanercept and Minocycline in a rat model of spinal cord injury. *European Journal of Pain*.

[44] Lechpammer M., Manning S. M., Samonte F. (2008). Minocycline treatment following hypoxic/ischaemic injury attenuates white matter injury in a rodent model of periventricular leucomalacia. *Neuropathology and Applied Neurobiology*.

[45] Liao T. V., Forehand C. C., Hess D. C., Fagan S. C. (2013). Minocycline repurposing in critical illness: focus on stroke. *Current Topics in Medicinal Chemistry*.

[46] Chen Y., Won S. J., Xu Y., Swanson R. A. (2014). Targeting microglial activation in stroke therapy: pharmacological tools and gender effects. *Current Medicinal Chemistry*.

[47] Chiarugi A., Moskowitz M. A. (2003). Poly(ADP-ribose) polymerase-1 activity promotes NF-*κ*B-driven transcription and microglial activation: implication for neurodegenerative disorders. *Journal of Neurochemistry*.

[48] Kobayashi K., Imagama S., Ohgomori T. (2013). Minocycline selectively inhibits M1 polarization of microglia. *Cell Death & Disease*.

[49] Papa S., Caron I., Erba E. (2016). Early modulation of pro-inflammatory microglia by minocycline loaded nanoparticles confers long lasting protection after spinal cord injury. *Biomaterials*.

[50] Burke N. N., Kerr D. M., Moriarty O., Finn D. P., Roche M. (2014). Minocycline modulates neuropathic pain behaviour and cortical M1-M2 microglial gene expression in a rat model of depression. *Brain, Behavior, and Immunity*.

[51] Yang Y., Salayandia V. M., Thompson J. F., Yang L. Y., Estrada E. Y., Yang Y. (2015). Attenuation of acute stroke injury in rat brain by minocycline promotes blood-brain barrier remodeling and alternative microglia/macrophage activation during recovery. *Journal of Neuroinflammation*.

[52] Francis J., Chu Y., Johnson A. K., Weiss R. M., Felder R. B. (2004). Acute myocardial infarction induces hypothalamic cytokine synthesis. *American Journal of Physiology—Heart and Circulatory Physiology*.

[53] Cheong C.-U., Chang C.-P., Chao C.-M., Cheng B.-C., Yang C.-Z., Chio C.-C. (2013). Etanercept attenuates traumatic brain injury in rats by reducing brain TNF-*α* contents and by stimulating newly formed neurogenesis. *Mediators of Inflammation*.

[54] Chio C.-C., Chang C.-H., Wang C.-C. (2013). Etanercept attenuates traumatic brain injury in rats by reducing early microglial expression of tumor necrosis factor-*α*. *BMC Neuroscience*.

[55] Zhang H.-W., Liu H.-Y., Wang B., Zhu Z.-Q. (2014). Efficacy of delayed administration of etanercept after spinal cord injury. *Journal of Peking University. Health Sciences*.

[56] Bayrakli F., Balaban H., Ozum U., Duger C., Topaktas S., Kars H. Z. (2012). Etanercept treatment enhances clinical and neuroelectrophysiological recovery in partial spinal cord injury. *European Spine Journal*.

[57] Clausen B. H., Degn M., Martin N. A. (2014). Systemically administered anti-TNF therapy ameliorates functional outcomes after focal cerebral ischemia. *Journal of Neuroinflammation*.

[58] Arango-Dávila C. A., Vera A., Londoño A. C. (2015). Soluble or soluble/membrane TNF-*α* inhibitors protect the brain from focal ischemic injury in rats. *International Journal of Neuroscience*.

[59] Tobinick E., Kim N. M., Reyzin G., Rodriguez-Romanacce H., Depuy V. (2012). Selective TNF inhibition for chronic stroke and traumatic brain injury: an observational study involving 629 consecutive patients treated with perispinal etanercept. *CNS Drugs*.

[60] Armitage J. (2007). The safety of statins in clinical practice. *The Lancet*.

[61] Charidimou A., Merwick Á. (2016). Statin therapy in acute ischemic stroke: time for large randomized trials?. *Neurology*.

[62] Lin F.-C., Chuang Y.-S., Hsieh H.-M. (2015). Early statin use and the progression of Alzheimer disease: a total population-based case-control study. *Medicine*.

[63] Bai S., Song Y., Huang X. (2016). Statin use and the risk of Parkinson's disease: an updated meta-analysis. *PLoS ONE*.

[64] Li T., Wang D., Tian Y. (2014). Effects of atorvastatin on the inflammation regulation and elimination of subdural hematoma in rats. *Journal of the Neurological Sciences*.

[65] Li B., Mahmood A., Lu D. (2009). Simvastatin attenuates microglial cells and astrocyte activation and decreases interleukin-1*β* level after traumatic brain injury. *Neurosurgery*.

[66] Wu H., Lu D., Jiang H. (2008). Increase in phosphorylation of Akt and its downstream signaling targets and suppression of apoptosis by simvastatin after traumatic brain injury. *Journal of Neurosurgery*.

[67] Wu H., Jiang H., Lu D. (2011). Induction of angiogenesis and modulation of vascular endothelial growth factor receptor-2 by simvastatin after traumatic brain injury. *Neurosurgery*.

[68] Kata D., Földesi I., Feher L. Z., Hackler L., Puskas L. G., Gulya K. (2016). Rosuvastatin enhances anti-inflammatory and inhibits pro-inflammatory functions in cultured microglial cells. *Neuroscience*.

[69] Chen X.-Y., Li K., Light A. R., Fu K.-Y. (2013). Simvastatin attenuates formalin-induced nociceptive behaviors by inhibiting microglial RhoA and p38 MAPK activation. *Journal of Pain*.

[70] Li Q.-Z., Sun J., Han J.-J., Qian Z.-J. (2013). Anti-inflammation of simvastatin by polarization of murine macrophages from M1 phenotype to M2 phenotype. *National Medical Journal of China*.

[71] Yang N., Cheng W., Hu H. (2016). Atorvastatin attenuates sympathetic hyperinnervation together with the augmentation of M2 macrophages in rats postmyocardial infarction. *Cardiovascular Therapeutics*.

[72] Zhang O., Zhang J. (2015). Atorvastatin promotes human monocyte differentiation toward alternative M2 macrophages through p38 mitogen-activated protein kinase-dependent peroxisome proliferator-activated receptor *γ* activation. *International Immunopharmacology*.

[73] Baur J. A., Sinclair D. A. (2006). Therapeutic potential of resveratrol: the in vivo evidence. *Nature Reviews Drug Discovery*.

[74] Lopez M. S., Dempsey R. J., Vemuganti R. (2015). Resveratrol neuroprotection in stroke and traumatic CNS injury. *Neurochemistry International*.

[75] Wang L., Wang Y., Cui M. (2016). A dietary polyphenol resveratrol acts to provide neuroprotection in recurrent stroke models by regulating AMPK and SIRT1 signaling, thereby reducing energy requirements during ischemia. *European Journal of Neuroscience*.

[76] Tellone E., Galtieri A., Russo A., Giardina B., Ficarra S. (2015). Resveratrol: a focus on several neurodegenerative diseases. *Oxidative Medicine and Cellular Longevity*.

[77] Feng Y., Cui Y., Gao J. L. (2016). Neuroprotective effects of resveratrol against traumatic brain injury in rats: involvement of synaptic proteins and neuronal autophagy. *Molecular Medicine Reports*.

[78] Shao A.-W., Wu H.-J., Chen S., Ammar A.-B., Zhang J.-M., Hong Y. (2014). Resveratrol attenuates early brain injury after subarachnoid hemorrhage through inhibition of NF-*κ*B-dependent inflammatory/MMP-9 pathway. *CNS Neuroscience and Therapeutics*.

[79] Feng Y., Cui Y., Gao J.-L. (2016). Resveratrol attenuates neuronal autophagy and inflammatoryinjury by inhibiting the TLR4/NF-*κ*B signaling pathwayin experimental traumatic brain injury. *International Journal of Molecular Medicine*.

[80] Zhang Q., Yuan L., Zhang Q. (2015). Resveratrol attenuates hypoxia-induced neurotoxicity through inhibiting microglial activation. *International Immunopharmacology*.

[81] Gatson J. W., Liu M.-M., Abdelfattah K. (2013). Resveratrol decreases inflammation in the brain of mice with mild traumatic brain injury. *Journal of Trauma and Acute Care Surgery*.

[82] Girbovan C., Plamondon H. (2015). Resveratrol downregulates type-1 glutamate transporter expression and microglia activation in the hippocampus following cerebral ischemia reperfusion in rats. *Brain Research*.

[83] Li L., Sun Q., Li Y. (2015). Overexpression of SIRT1 induced by resveratrol and inhibitor of miR-204 suppresses activation and proliferation of microglia. *Journal of Molecular Neuroscience*.

[84] Wang F., Cui N., Yang L. (2015). Resveratrol rescues the impairments of hippocampal neurons stimulated by microglial over-activation in vitro. *Cellular and Molecular Neurobiology*.

[85] Dragone T., Cianciulli A., Calvello R., Porro C., Trotta T., Panaro M. A. (2014). Resveratrol counteracts lipopolysaccharide-mediated microglial inflammation by modulating a SOCS-1 dependent signaling pathway. *Toxicology in Vitro*.

[86] Kodali M., Parihar V. K., Hattiangady B., Mishra V., Shuai B., Shetty A. K. (2015). Resveratrol prevents age-related memory and mood dysfunction with increased hippocampal neurogenesis and microvasculature, and reduced glial activation. *Scientific Reports*.

[87] Porro C., Cianciulli A., Calvello R., Panaro M. A. (2015). Reviewing the role of resveratrol as a natural modulator of microglial activities. *Current Pharmaceutical Design*.

[88] Song J., Cheon S. Y., Jung W., Lee W. T., Lee J. E. (2014). Resveratrol induces the expression of interleukin-10 and brain-derived neurotrophic factor in BV2 microglia under hypoxia. *International Journal of Molecular Sciences*.

[89] Cianciulli A., Dragone T., Calvello R. (2015). IL-10 plays a pivotal role in anti-inflammatory effects of resveratrol in activated microglia cells. *International Immunopharmacology*.

[90] Zhang Z.-Y., Sun B.-L., Liu J.-K. (2015). Activation of mGluR5 attenuates microglial activation and neuronal apoptosis in early brain injury after experimental subarachnoid hemorrhage in rats. *Neurochemical Research*.

[91] Byrnes K. R., Loane D. J., Stoica B. A., Zhang J., Faden A. I. (2012). Delayed mGluR5 activation limits neuroinflammation and neurodegeneration after traumatic brain injury. *Journal of Neuroinflammation*.

[92] Loane D. J., Stoica B. A., Byrnes K. R., Jeong W., Faden A. I. (2013). Activation of mGluR5 and inhibition of NADPH oxidase improves functional recovery after traumatic brain injury. *Journal of Neurotrauma*.

[93] Qiu J.-L., Zhu W.-L., Lu Y.-J. (2015). The selective mGluR5 agonist CHPG attenuates SO2-induced oxidative stress and inflammation through TSG-6/NF-kappaB pathway in BV2 microglial cells. *Neurochemistry International*.

[94] Byrnes K. R., Stoica B., Loane D. J., Riccio A., Davis M. I., Faden A. I. (2009). Metabotropic glutamate receptor 5 activation inhibits microglial associated inflammation and neurotoxicity. *GLIA*.

[95] Loane D. J., Stoica B. A., Tchantchou F. (2014). Novel mGluR5 positive allosteric modulator improves functional recovery, attenuates neurodegeneration, and alters microglial polarization after experimental traumatic brain injury. *Neurotherapeutics*.

[96] Park L., Anrather J., Girouard H., Zhou P., Iadecola C. (2007). Nox2-derived reactive oxygen species mediate neurovascular dysregulation in the aging mouse brain. *Journal of Cerebral Blood Flow and Metabolism*.

[97] Girouard H., Park L., Anrather J., Zhou P., Iadecola C. (2006). Angiotensin II attenuates endothelium-dependent responses in the cerebral microcirculation through nox-2-derived radicals. *Arteriosclerosis, Thrombosis, and Vascular Biology*.

[98] Khayrullina G., Bermudez S., Byrnes K. R. (2015). Inhibition of NOX2 reduces locomotor impairment, inflammation, and oxidative stress after spinal cord injury. *Journal of Neuroinflammation*.

[99] Savchenko V. L. (2013). Regulation of NADPH oxidase gene expression with PKA and cytokine IL-4 in neurons and microglia. *Neurotoxicity Research*.

[100] Choi S.-H., Aid S., Kim H.-W., Jackson S. H., Bosetti F. (2012). Inhibition of NADPH oxidase promotes alternative and anti-inflammatory microglial activation during neuroinflammation. *Journal of Neurochemistry*.

[101] Zhang Q.-G., Laird M. D., Han D. (2012). Critical role of nadph oxidase in neuronal oxidative damage and microglia activation following traumatic brain injury. *PLoS ONE*.

[102] Kumar A., Barrett J. P., Alvarez-Croda D., Stoica B. A., Faden A. I., Loane D. J. (2016). NOX2 drives M1-like microglial/macrophage activation and neurodegeneration following experimental traumatic brain injury. *Brain, Behavior, and Immunity*.

[103] Han L., Cai W., Mao L. (2015). Rosiglitazone promotes white matter integrity and long-term functional recovery after focal cerebral ischemia. *Stroke*.

[104] Liu H., Rose M. E., Culver S., Ma X., Dixon C. E., Graham S. H. (2016). Rosiglitazone attenuates inflammation and CA3 neuronal loss following traumatic brain injury in rats. *Biochemical and Biophysical Research Communications*.

[105] Gu C., Wang Y., Li J. (2015). Rosiglitazone attenuates early brain injury after experimental subarachnoid hemorrhage in rats. *Brain Research*.

[106] Pisanu A., Lecca D., Mulas G. (2014). Dynamic changes in pro- and anti-inflammatory cytokines in microglia after PPAR-*γ* agonist neuroprotective treatment in the MPTPp mouse model of progressive Parkinson's disease. *Neurobiology of Disease*.

[107] Mandrekar-Colucci S., Karlo J. C., Landreth G. E. (2012). Mechanisms underlying the rapid peroxisome proliferator-activated receptor-*γ*-mediated amyloid clearance and reversal of cognitive deficits in a murine model of Alzheimer's disease. *The Journal of Neuroscience*.

[108] Inaba T., Katayama Y., Ueda M., Nito C. (2015). Neuroprotective effects of pretreatment with macrolide antibiotics on cerebral ischemia reperfusion injury. *Neurological Research*.

[109] Murphy B. S., Sundareshan V., Cory T. J., Hayes D., Anstead M. I., Feola D. J. (2008). Azithromycin alters macrophage phenotype. *Journal of Antimicrobial Chemotherapy*.

[110] Hodge S., Hodge G., Jersmann H. (2008). Azithromycin improves macrophage phagocytic function and expression of mannose receptor in chronic obstructive pulmonary disease. *American Journal of Respiratory and Critical Care Medicine*.

[111] Vrančić M., Banjanac M., Nujić K. (2012). Azithromycin distinctively modulates classical activation of human monocytes in vitro. *British Journal of Pharmacology*.

[112] Amantea D., Certo M., Petrelli F., Bagetta G. (2016). Neuroprotective properties of a macrolide antibiotic in a mouse model of middle cerebral artery occlusion: characterization of the immunomodulatory effects and validation of the efficacy of intravenous administration. *Assay and Drug Development Technologies*.

[113] Amantea D., Certo M., Petrelli F. (2016). Azithromycin protects mice against ischemic stroke injury by promoting macrophage transition towards M2 phenotype. *Experimental Neurology*.

[114] Zhang B., Bailey W. M., Kopper T. J., Orr M. B., Feola D. J., Gensel J. C. (2015). Azithromycin drives alternative macrophage activation and improves recovery and tissue sparing in contusion spinal cord injury. *Journal of Neuroinflammation*.

[115] Verbois S. L., Scheff S. W., Pauly J. R. (2003). Chronic nicotine treatment attenuates *α*7 nicotinic receptor deficits following traumatic brain injury. *Neuropharmacology*.

[116] Duris K., Manaenko A., Suzuki H., Rolland W. B., Krafft P. R., Zhang J. H. (2011). *α*7 nicotinic acetylcholine receptor agonist PNU-282987 attenuates early brain injury in a perforation model of subarachnoid hemorrhage in rats. *Stroke*.

[117] Dash P. K., Zhao J., Kobori N. (2016). Activation of alpha 7 cholinergic nicotinic receptors reduce blood–brain barrier permeability following experimental traumatic brain injury. *Journal of Neuroscience*.

[118] Han Z., Li L., Wang L., Degos V., Maze M., Su H. (2014). Alpha-7 nicotinic acetylcholine receptor agonist treatment reduces neuroinflammation, oxidative stress, and brain injury in mice with ischemic stroke and bone fracture. *Journal of Neurochemistry*.

[119] Han Z., Shen F., He Y. (2014). Activation of *α*-7 nicotinic acetylcholine receptor reduces ischemic stroke injury through reduction of pro-inflammatory macrophages and oxidative stress. *PLoS ONE*.

[120] Tehranian R., Andell-Jonsson S., Beni S. M. (2002). Improved recovery and delayed cytokine induction after closed head injury in mice with central overexpression of the secreted isoform of the interleukin-1 receptor antagonist. *Journal of Neurotrauma*.

[121] Relton J. K., Rothwell N. J. (1992). Interleukin-1 receptor antagonist inhibits ischaemic and excitotoxic neuronal damage in the rat. *Brain Research Bulletin*.

[122] Clausen F., Hånell A., Björk M. (2009). Neutralization of interleukin-1*β* modifies the inflammatory response and improves histological and cognitive outcome following traumatic brain injury in mice. *European Journal of Neuroscience*.

[123] Clausen F., Hånell A., Israelsson C. (2011). Neutralization of interleukin-1*β* reduces cerebral edema and tissue loss and improves late cognitive outcome following traumatic brain injury in mice. *European Journal of Neuroscience*.

[124] Helmy A., Guilfoyle M. R., Carpenter K. L. H., Pickard J. D., Menon D. K., Hutchinson P. J. (2014). Recombinant human interleukin-1 receptor antagonist in severe traumatic brain injury: a phase II randomized control trial. *Journal of Cerebral Blood Flow and Metabolism*.

[125] Helmy A., Guilfoyle M. R., Carpenter K. L. H., Pickard J. D., Menon D. K., Hutchinson P. J. (2016). Recombinant human interleukin-1 receptor antagonist promotes M1 microglia biased cytokines and chemokines following human traumatic brain injury. *Journal of Cerebral Blood Flow and Metabolism*.

[126] Gao J., Prough D. S., McAdoo D. J. (2006). Transplantation of primed human fetal neural stem cells improves cognitive function in rats after traumatic brain injury. *Experimental Neurology*.

[127] Mosher K. I., Andres R. H., Fukuhara T. (2012). Neural progenitor cells regulate microglia functions and activity. *Nature Neuroscience*.

[128] Liu J., Hjorth E., Zhu M. (2013). Interplay between human microglia and neural stem/progenitor cells in an allogeneic co-culture model. *Journal of Cellular and Molecular Medicine*.

[129] Gao J., Grill R. J., Dunn T. J. (2016). Human neural stem cell transplantation-mediated alteration of microglial/macrophage phenotypes after traumatic brain injury. *Cell Transplantation*.

[130] Walker P. A., Bedi S. S., Shah S. K. (2012). Intravenous multipotent adult progenitor cell therapy after traumatic brain injury: modulation of the resident microglia population. *Journal of Neuroinflammation*.

